# Reverse Rim Sign

**DOI:** 10.5334/jbsr.1462

**Published:** 2018-02-14

**Authors:** Thomas Thuysbaert, Chloë Standaert, Pieter De Visschere

**Affiliations:** 1 Ghent University Hospital, BE

**Keywords:** CT, Acute cortical necrosis, Shock, Kidney, Renal failure

## Case History

A 41-year-old male with a history of acute myeloid leukemia (AML) presenting with a pneumonia and neutropenic fever was admitted to the intensive care unit with symptoms of shock.

**Figure 1:**
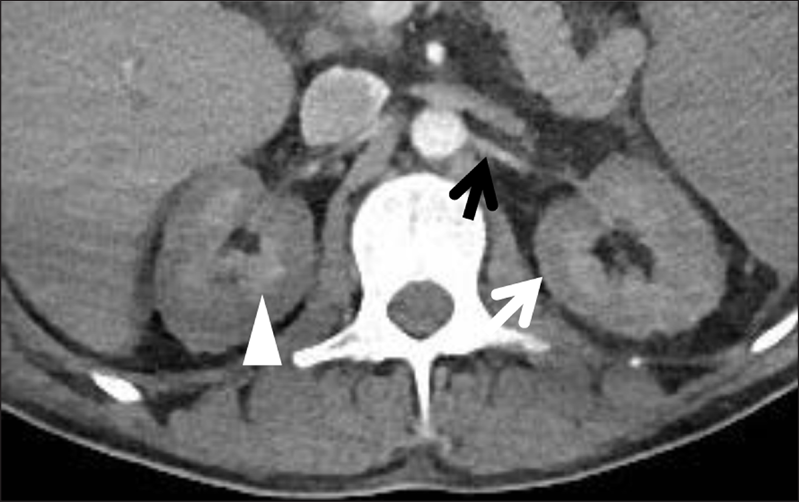
Axial contrast enhanced CT in the arterial phase shows a non-enhancing renal cortex with a capsular enhancing rim (white arrow) and enhancement of the renal medulla (arrowhead). Also note the fine caliber of the renal artery (black arrow).

**Figure 2:**
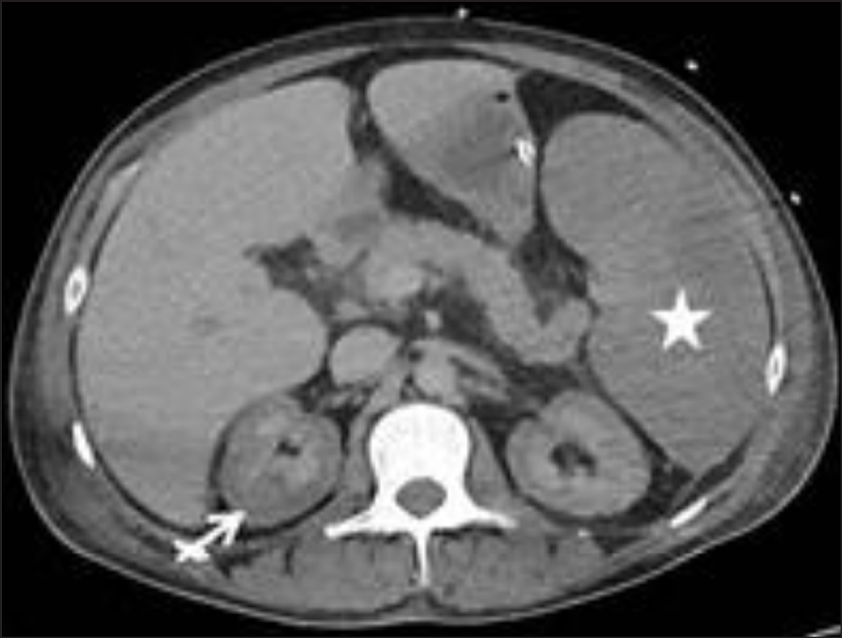
Axial contrast-enhanced CT in the venous phase showing the same renal enhancement pattern as in the arterial phase with a non enhancing renal cortex (white arrow). Also note splenomegaly (asterisk).

**Figure 3:**
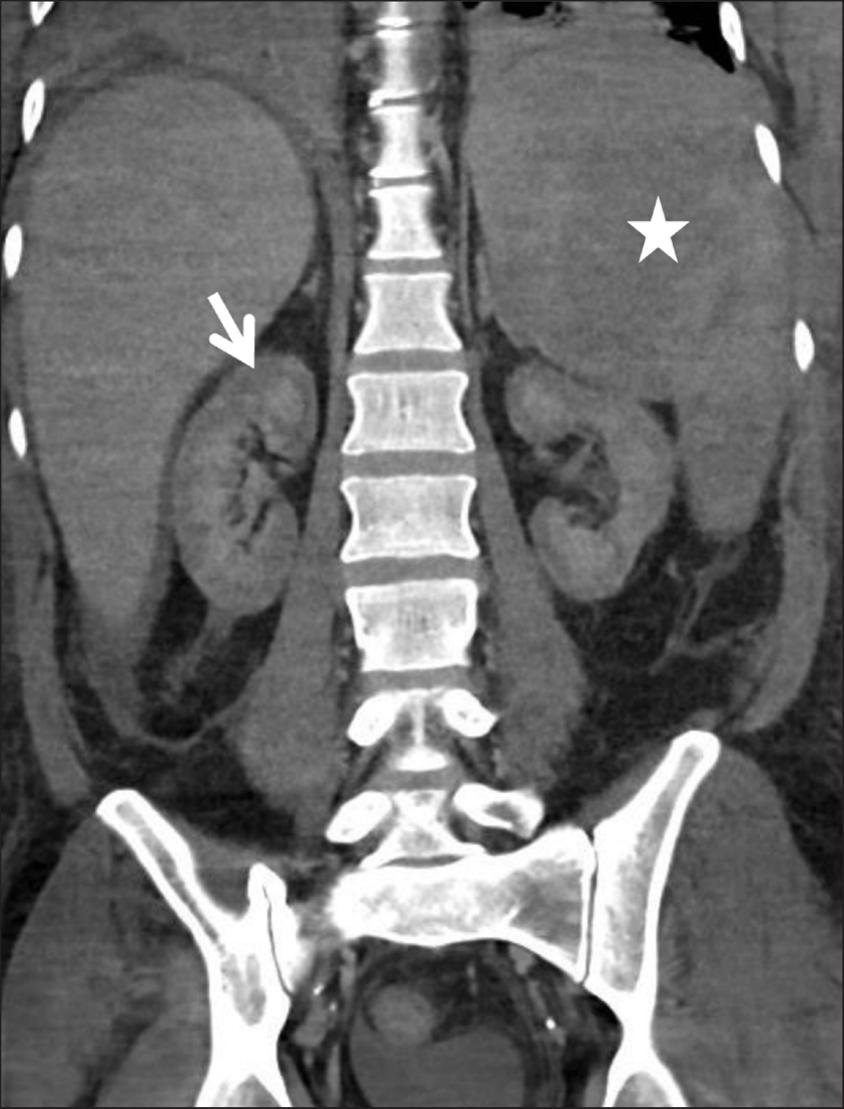
Coronal contrast-enhanced CT in the venous phase shows non enhancing renal cortex (white arrow). Also note splenomegaly (asterisk) and the small amount of perihepatic ascites.

An urgent contrast-enhanced computed tomography (CT) was performed to exclude active bleeding (Figure 1, arterial phase; Figures 2 and 3, venous phase). The CT showed enhancement of the renal medulla (Figure 1, arrowhead), hypoattenuation of the renal cortex and a small rim of cortical enhancement in the arterial and venous phase (Figures 1 and 3, white arrow). These findings were compatible with acute cortical necrosis. A fine caliber of the renal arteries and the hypoattenuating liver and spleen due to shock were also noted (Figure 1, black arrow). Also note the splenomegaly due to AML (Figure 3, asterisk). No signs of active bleeding were found. At the time of the CT scan, the most recent renal function was not yet known. Lab results shortly after the scan showed acute renal failure.

## Comment

Renal acute cortical necrosis (ACN) is a rare form of acute renal failure presenting as ischemic necrosis of the renal cortex with sparing of the renal medulla. ACN is typically seen in conditions that produce acute and prolonged shock such as severe hemorrhage, trauma, dehydration or as in this case due to neutropenic shock [1]. ACN is bilateral in most cases. The underlying physiopathology includes vasoconstriction of small intracortical blood vessels, damaged glomerular endothelium and intravascular thrombosis.

ACN gives a typical renal enhancement pattern on contrast-enhanced CT imaging. In arterial phase the CT scan shows enhancement of the interlobular and arcuate arteries adjacent to the non-enhancing cortex. In the portovenous phase, the CT shows enhancement of the renal medulla with a hypoattenuating, non-enhancing cortex; this is called the “reverse rim sign”. A thin millimetric rim of capsular and cortical enhancement can be visible due to the presence of collateral flow from the capsular blood supply. This is referred to as the “cortical rim sign”.

In chronic stages the kidneys become small and a rim or tramline calcification in the cortex can be formed two months after the initial event. ACN is often irreversible, and no specific treatment is recommended besides supportive therapy.

## References

[1] Kawashima, A, Sandler, CM, Ernst, RD, et al. CT evaluation of renovascular disease. Radiographics. 2000; 20: 1321–1340. DOI: 10.1148/radiographics.20.5.g00se141321 10992021

